# Current and Future Experimental Strategies for Structural Analysis of Trichothecene Mycotoxins-A Prospectus

**DOI:** 10.3390/toxins3121518

**Published:** 2011-12-19

**Authors:** Roxanne A. Shank, Nora A. Foroud, Paul Hazendonk, François Eudes, Barbara A. Blackwell

**Affiliations:** 1 Agriculture and Agri-Food Canada, 5403 1 Ave S, Lethbridge, AB T1J 4B1, Canada; Email: shankr2@uleth.ca (R.A.S.); nora.foroud@agr.gc.ca (N.A.F.); 2 University of Lethbridge, 4401 University Dr W, Lethbridge, AB T1K 3M4, Canada; 3 Agriculture and Agri-Food Canada, 930 Carling Ave, Ottawa, ON K1A 0C5, Canada; Email: barbara.blackwell@agr.gc.ca

**Keywords:** trichothecene, NMR, solid-state NMR, crystallography, NMR crystallography, natural product, mycotoxin, structure, structure-function, molecular modeling, molecular dynamics, antibiotic, ribosome

## Abstract

Fungal toxins, such as those produced by members of the order *Hypocreales*, have widespread effects on cereal crops, resulting in yield losses and the potential for severe disease and mortality in humans and livestock. Among the most toxic are the trichothecenes. Trichothecenes have various detrimental effects on eukaryotic cells including an interference with protein production and the disruption of nucleic acid synthesis. However, these toxins can have a wide range of toxicity depending on the system. Major differences in the phytotoxicity and cytotoxicity of these mycotoxins are observed for individual members of the class, and variations in toxicity are observed among different species for each individual compound. Furthermore, while diverse toxicological effects are observed throughout the whole cellular system upon trichothecene exposure, the mechanism of toxicity is not well understood. In order to comprehend how these toxins interact with the cell, we must first have an advanced understanding of their structure and dynamics. The structural analysis of trichothecenes was a subject of major interest in the 1980s, and primarily focused on crystallographic and solution-state Nuclear Magnetic Resonance (NMR) spectroscopic studies. Recent advances in structural determination through solution- and solid-state NMR, as well as computation based molecular modeling is leading to a resurgent interest in the structure of these and other mycotoxins, with the focus shifting in the direction of structural dynamics. The purpose of this work is to first provide a brief overview of the structural data available on trichothecenes and a characterization of the methods commonly employed to obtain such information. A summary of the current understanding of the relationship between structure and known function of these compounds is also presented. Finally, a prospectus on the application of new emerging structural methods on these and other related systems is discussed.

## 1. Introduction

The search for biologically active natural products in bacteria, fungi, and higher plants has been a source of major breakthroughs, particularly in medicine. Many small molecules found in nature have provided inspiration to the pharmaceutical industry for the development of new and more effective drugs based on their structures, while others have led to the discovery of toxic compounds which may contribute to life-threatening diseases. The discovery of such toxic molecules becomes important when considering disease prevention. Among the compounds which have been isolated in the past thirty years are a group of fungal toxins (mycotoxins) known as trichothecenes. Trichothecenes are produced by a range of fungi from the order *Hypocreales*, including those of the genera *Fusarium*, *Myrothecium*, *Verticimonosporium*, *Stachybotrys*, *Trichoderma*, *Trichothecium*, *Cephalosporium*, and *Cylindrocarpon* [1,2,3,4,5]. Although the majority of trichothecenes contribute to crop disease and mycotoxicoses, they have also been considered as antibiotics and antileukemics [5,6,7,8]. In order to better understand the function of trichothecenes, to prevent diseases associated with these toxins (*i.e.*, Fusarium Head Blight (FHB) of grains and stachybotryotoxicosis in mammals), and alternatively explore the roles they may play as powerful pharmaceutical agents, it is important to gain insight into the biochemical processes and structure of this important class of compounds.

Trichothecene-producing fungi were originally discovered as contributors to mold in grain products as early as the 1930s and 1940s [9]. The *Fusarium* and *Stachybotrys* genera are frequently associated with the infection of crops in temperate climates, such as Europe, Asia and the Americas. *Stachybotrys* is a saprophytic fungus, which is commonly found to infect high-cellulosic crops such as straw and hay, and is the leading cause of stachybotryotoxicosis in livestock [10]. Furthermore, *Stachybotrys* is a toxic mold commonly found in association with sick building syndrome; a multitude of illnesses which are associated with poor air quality in office buildings [11]. *Fusarium* species are responsible for a wide variety of plant diseases, including fusarium head blight (FHB) and crown rot in cereal crops (*i.e.*, barley, wheat, rye, *etc*.) [12,13] and fusarium wilt of solanaceous crops (*i.e.*, potato, tomato, *etc*.) [14,15]. These diseases can result in severe yield loss in susceptible crops. In the case of FHB, trichothecenes accumulate in the developing grain of cereal crops. Ingestion of trichothecene-contaminated grain has been linked to emesis, hemorrhaging, abortion and death in animals (reviewed in [16]). In humans, ingestion of trichothecenes is the leading cause of alimentary toxic aleukia, a condition characterized by vomiting, diarrhea, anemia, dermatitis, gastrointestinal necrosis and can be lethal, particularly in immune-suppressed persons [17]. 

The first trichothecene to be isolated was trichothecin from *Trichotheceum roseum*, in 1948 by Freeman and Morrison [18]. Diacetoxyscirpenol (DAS) from *Fusarium equiseti* was preliminarily characterized in 1961 by Brian *et al.* [xref^19^], and was later followed by nivalenol (NIV) [20] and T-2 toxin [21], both from *F. sporotrichioides*, although they were mis-identified as *F. nivale* and *F. tricincum*, respectively, in the original articles [22]. However, it was the discovery of 4-deoxynivalenol (DON) from wheat in Eastern North America in 1980 [23], which truly sparked the research into the *Fusarium* species and led to the discovery of trichothecenes from other genera.

**Figure 1:**
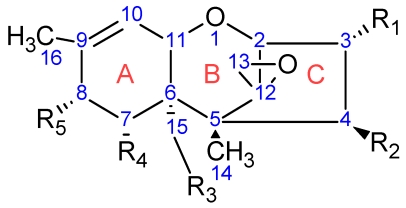
Chemical structure of the trichothecene core. Substituents R1 through R5 are depicted with their stereochemical configuration off the core.

**Figure 2:**
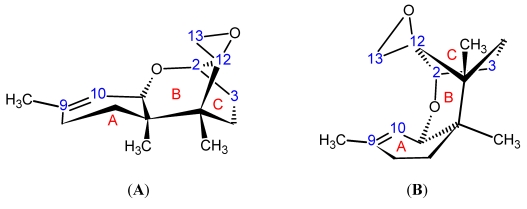
Three-dimensional stereochemistry of the trichothecene core when (**A**) the A-ring is in a half-chair, and the B-ring in a chair conformation; and (**B**) the A-ring is a half-chair, and the B-ring in a boat conformation.

Trichothecenes are a large group of sesquiterpenoid fungal metabolites, which share a common core comprised of a rigid tetracyclic ring system (Figure 1) consisting of a cyclohexene, A-ring with a double C-C bond occurring between C-9 and C-10; a tetrahydropyranyl B-ring; a cyclopentyl C-ring, and an epoxide at C-12/13. The rigidity of this system results in a distinct stereochemistry for the A- and B-rings. The A-ring adopts a half-chair conformation, and the B-ring is most often found in the chair conformation (Figure 2A) [24,25], although there have been a few odd cases where the B-ring has been shown to adopt a boat conformation (Figure 2B) [26].

**Figure 3:**
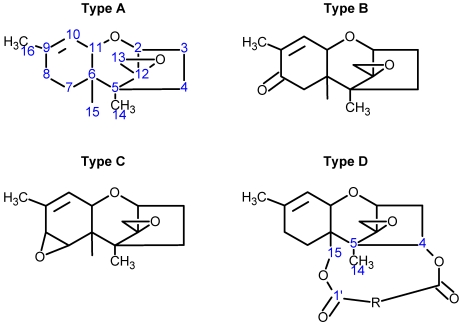
The general core structures for Type A, B, C, and D trichothecenes.

Over 200 trichothecene compounds have been isolated, and they fall into two main classes, simple and macrocyclic [2,27,28]. The simple trichothecenes are further divided into three types; A, B and C. Type A trichothecenes are the simplest group, being non-substituted, hydroxylated or esterified (Figure 3). Type B trichothecenes are characterized by a ketone present at C-8 (Figure 3) Type A and B trichothecenes, such as T-2 toxin and DON, respectively, are often associated with *Fusarium*-infected grain. Type C trichothecenes, such as crotocin [29], are less common than the others, and are distinguished by the presence of a second epoxide ring at C-7/8 (Figure 3). A fourth class (Type D), are characterized by the presence of a cyclic diester or triester linkage of C-4 to C-15 (Figure 3) [30]. These macrocyclic trichothecenes include the satratoxins, verrucarins, roridins, myrotoxins and baccharinoids (Table 1). While many of the Type D trichothecenes have been isolated from fungi, the baccharinoids were first isolated from the plant *Baccharis* [31]. Although early reports suggested that the metabolites were produced by the plant itself, later studies indicated that the toxins were likely the product of a *Hypocrealean* endophyte (the order to which trichothecene-producing fungi such as *Fusarium*, *Myrothecium* and *Stachybotrys* belong) within the plants [32]. It is important to note here that there are other secondary metabolites produced by trichothecene-producing fungi which may have relevance in the pathogenenicity of some diseases, such as FHB. Some of these metabolites are derived from the same trichodiene precursor molecule as trichothecenes, but are products of different cyclizations [33,34,35]. Together with the trichothecenes, these molecules belong to the trichodienoid class of compounds. The non-trichothecene trichodienoids do not possess the C-13 epoxide ring system essential for known mechanisms of toxicity. 

**Table 1:** Chemical substitutions of some common trichothecenes. R groups refer to substituents shown in Figure 1.

Toxin Name	Type	R1	R2	R3	R4	R5
T-2 toxin	A	OH	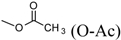 (O-Ac)	O-Ac	H	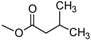
Diacetoxyscirpenol (DAS)	A	OH	O-Ac	O-Ac	H	H
Trichodermin	A	H	O-Ac	H	H	H
Decalonectrin	A	O-Ac	H	OH	H	H
Deoxynivalenol (DON)	B	OH	H	OH	OH	=O
3-acetyldeoxynivalenol (3-ADON)	B	O-Ac	H	OH	OH	=O
Nivalenol (NIV)	B	OH	OH	OH	OH	=O
Trichothecin	B	H	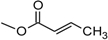	H	H	=O
Crotocin	C	H	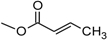	H	Epoxide	
Isororidin A	D	H	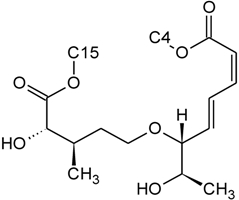		H	H
Roridin E	D	H	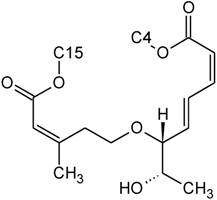		H	H
Roridin H (Verrucarin H)	D	H	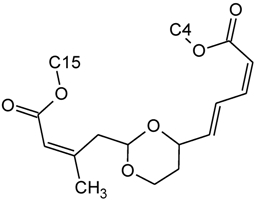		H	H
Baccharinoid B1	D	H	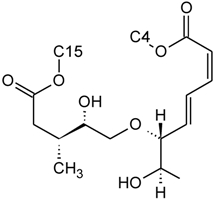		H	H
Baccharinoid B2	D	H	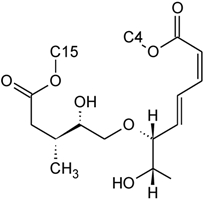		H	H
Baccharinoid B3	D	H	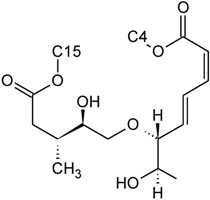		H	H
Baccharinoid B7	D	H	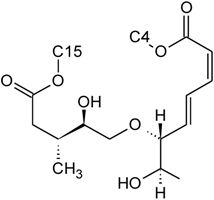		H	H
Verrucarin A	D	H	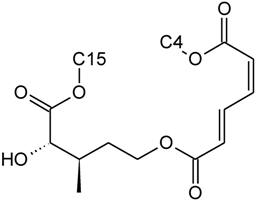		H	H
Verrucarin B	D	H	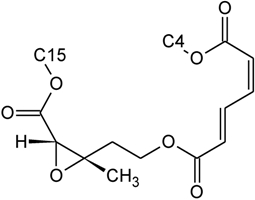		H	H
Myrotoxin A	D	H	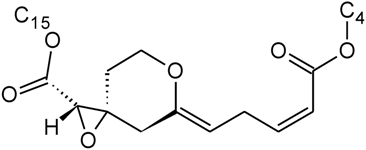		H	H
Myrotoxin B	D	H	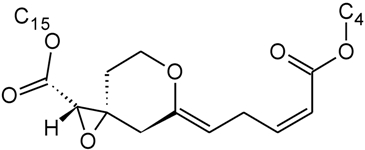		H	OH

Trichothecenes have widespread toxicological effects throughout the cell, and have been implicated in membrane destabilization, cytoskeletal collapse, inhibition of RNA and DNA synthesis, inhibition of mitochondrial function, and induction of apoptosis [36]. However, the best studied effect of the trichothecene toxins has been inhibition of protein synthesis through an interaction with eukaryotic ribosomes [37,38,39,40]. This targeting of the ribosome is similar to the interaction of mainstream antibiotics with prokaryotic ribosomes, and it is for this reason that members of this class of toxins have been referred to as eukaryote-specific antibiotics [6,41]. Since the epoxide ring is necessary for disruption of protein synthesis [42], it is logical to estimate that the epoxide plays a significant role in the inhibitory mechanism. 

There has been much interest in the differences in relative toxicity observed among this class of compounds. Species-specific differences in toxicity of a given trichothecene have been observed not only between plants and animals, but more interestingly among different plant species (e.g., corn *vs.* rice) [43], and among different animal species (e.g., monkeys *vs.* mice) [44]. A better understanding of the structural differences leading to the variable toxicity observed among various species may help scientists to develop antifungal and antiparasitic compounds with little to no toxic effects on the host organism. It is important to mention that very few bacteria are sensitive to trichothecenes and the few systems that do exhibit trichothecene susceptibility appear to be unrelated [45,46]. Interestingly, some probiotic strains of *Bacillus* have been shown to detoxify DON by opening the epoxide ring [47]. Regardless, relatively few toxicological effects have been observed for trichothecenes when tested on bacterial systems in comparison to eukaryotic strains, suggesting a certain degree of specificity for higher systems. Investigations regarding the cause of this discrimination between prokaryotic and eukaryotic systems are sparse, and it remains to be seen whether the toxicological resistance observed for prokaryotic systems is due to differences in cellular machinery, rapid metabolism, or inefficient membrane translocation. 

Furthermore, some research from the 1980s suggested that macrocylic trichothecenes target cancer cells and may be useful as antileukemics [4,7,8,48]. However, the mechanism of action of the trichothecene compounds, with regard to any of their toxicological effects is not well understood. Therefore, the absolute stereochemistry and structural configuration of these compounds must be analyzed in order to understand what the possible interactions of these molecules may be. Structural analysis has been used to identify secondary metabolites produced in genetic and feeding experiments, and this approach has enabled the characterization of metabolic and biosynthetic pathways associated with trichothecenes [49,50,51,52,53,54,55,56]. Detailed studies on the structure and dynamics of these compounds can also be used to help elucidate the underlying mechanisms for differences in toxicity among these compounds [57].

In this paper we discuss the structure and dynamic nature of these compounds in order to gain a better understanding of the mechanisms of toxicity of these small molecules. Furthermore, the methods for determining trichothecene structure, and an analysis of how trichothecene structure is related to function and activity, will be discussed. This is not meant to serve as an exhaustive database of trichothecene structural parameters, for that the reader is directed to previous reviews [2,24,25,26]. Rather, this is meant to serve as a prospectus on the methods used to study the three-dimensional structure of members from this class of mycotoxins and its significance to biology, biochemistry and plant biotechnology.

## 2. Structure-Function Relationships

### Trichothecene Toxicity

The widespread toxicological effects of trichothecenes are still a matter of much interest to researchers in the fields of plant biotechnology, cell biology, food chemistry and biochemistry [12]. It is widely accepted that the major mode of action of trichothecene toxicosis is the inhibition of protein synthesis in eukaryotes [39], and while the exact mechanism of this inhibition is unknown, it has been demonstrated to involve interactions with the peptidyl transferase center (PTC) of the 60S ribosome. Yeast strains carrying a mutation involving a substitution at a key tryptophan residue to a cystein (W255C) in the ribosomal protein L3 (RPL3) have been shown to confer resistance to the trichothecene trichodermin [37,58,59]. Harris and Gleddie [60] demonstrated that a similar substitution (W258C) in a modified rice *Rpl3* gene enabled DON-tolerance in transgenic tobacco. However, results presented by Mitterbauer *et al*. [61], who expressed a W258C mutated tomato *Rpl3* gene in tobacco, suggest that copy number of the substituted *Rpl3* gene may determine the level of resistance observed. The described tryptophan to cystein substitutions occur in a universally conserved region known as the tryptophan-finger (W-finger), which was shown to play a critical role in coordinating the steps involved in protein translation [62,63]. All of these studies suggest that trichothecenes exhibit a stereospecific interaction with the ribosome. 

Mitterbauer *et al.* [xref^61^] observed several mutations in tomato RPL3, in addition to W258C, which confer trichothecene resistance. He proposed that differences that have been observed in toxin resistance among different species may be linked to different isoforms of RPL3 among these organisms. Different trichothecenes have been shown to inhibit different stages of protein translation, including inhibition of initiation (Type I inhibitors), elongation (Type E) or termination (Type T) [38,40,64,65]. The type of inhibition observed is related to the substitution pattern of the side chains. For example trichothecenes with an oxygen functionality at C-15, will typically confer Type I inhibition [65], whereas an -H or -OH group at C-4 confers Type T inhibition [40]. It is possible that different isoforms of RPL3 are more resistant to a specific type of inhibitor, and is likely linked to hydrogen-bonding interactions that the side chains of the toxin can form with the other protein and nucleic acid residues in the PTC.

Trichothecenes have been shown to activate the ribotoxic stress response leading to apoptosis in mammalian cells, a process mediated by protein kinase signaling cascades [66,67,68,69,70]. Other cytotoxic effects of trichothecenes have also been observed, including inhibition of nucleic acids synthesis [39] and cell division [71], destabilization of cellular membranes [72], and inhibition of mitochondrial function [73,74]. In all cases the exact mechanism of the described toxicity remains a mystery, due to a lack of understanding regarding the properties of these toxins and how they interact with the cell [36]. Without this knowledge it is impossible to determine the genes which must be targeted in order to confer resistance [43]. Furthermore, is not known whether these are secondary effects of ribosomal toxicity and/or apoptosis, or if there is a direct interaction between trichothecenes and other components of the cell. The inhibitory effect on the 60S ribosomal subunit produces a stress response in the cell, which could result in downstream inhibition of the remaining cellular machinery. In fact, when the tryptophan residue of the W-finger is mutated to another functional residue, a reduction or complete abolishment of trichothecene toxicity is observed, depending on the residue introduced, the isoform of RPL3, and the host species [62,63]. Such mutagenic effects suggest that the widespread cytotoxic effects of trichothecenes are due to downstream processes that result from the inhibition of protein synthesis. 

Different trichothecenes have been shown to have different levels of toxicity within a species, and there are also differences of individual toxins in different organisms. For example, while T-2 toxin was shown to be less phytotoxic in wheat than DON [75], it has also been shown to be more toxic than DON in *Arabidopsis* [76] and in mammalian systems [77]. Similarly, while DON is generally more phytotoxic than NIV [75,76,78,79,80,81], the latter has been shown to be more toxic in some mammalian systems [82,83]. Variations in trichothecene phytoxicity may explain, in part, the differences in aggressiveness observed among different trichothecene-producing phenotypes (chemotypes) of *Fusarium* species involved in plant disease [84]. For example, NIV-producing *Fusarium* species are less virulent than DON producers in causing FHB [80]. It has been observed that C-3 acetylation can reduce phytotoxicity of specific trichothecenes in *Arabidopsis*, *Chlamydomonas*, tobacco and rice [76,85,86,87]; whereas, in wheat seedling germination and coleoptile growth inhibition studies, DON and 3-ADON were generally shown to be equally phytotoxic [75]. Thus, it is difficult to predict how a particular toxin will be tolerated from one species to another. The observed differences in phytotoxicity among species may be related to structural differences in the PTC of ribosomes, as discussed above. Additionally, the differential ability of some plants to metabolize these toxins [88,89,90] could also explain differences regarding the involvement of trichothecenes in the transmittance of plant disease. 

The differences in toxicity observed between plant and animal systems ‘may be due to the cellular uptake of these toxins. To date, no cellular receptors for trichothecene uptake have been identified, and the mode of entry into the cell is unknown. However, it is important to note that trichothecenes are amphipathic molecules [91], which may infer an ability for these molecules to enter the cell through some mode of direct translocation, and the variable lipophilicity of the toxins would have an effect on access to the cell. Anderson *et al*. have shown that a decrease in toxicity could be observed for compounds containing more than two free hydroxyl groups [92]. If trichothecenes are able to translocate across the cellular membrane, the architectural and biochemical differences between plant and animal systems, such as lipid composition and the presence or absence of a cell wall would have an impact on their cellular uptake. The action of T-2 toxin on cell membrane function in animal cells has been monitored [72], with the presence of phosphatidylcholine as a membrane constituent influencing the action of T-2 toxin [93]. It has been suggested that trichothecene toxins may act together in a synergistic fashion in order to convey virulence [94,95]. However, without a comprehensive understanding of the mode of action of a single toxin, it is impossible to determine, or even predict how it might act synergistically with other similar toxins [94,95]. Studies have been conducted *in vitro* which suggest that there is indeed an interactive relationship between trichothecenes [94,95]. Furthermore, there are various non-trichothecene secondary metabolites derived from trichodiene (described in the introduction) which, although not toxic in their own right, may act as virulence factors in plant and animal pathogenesis [43].

The first major structure-activity study of a trichothecene was performed in 1969 by Grove and Mortimer following isolation and characterization of 4,15-diacetoxyscirpenol (DAS) [96]. This study was the first to clearly demonstrate that both the epoxide moiety and the structural arrangement of the sesquiterpenoid ring system were essential features in trichothecene toxicity. Subsequent structure-function studies of trichothecenes have focused on how different substitution patterns affect toxicity [43,97]. 

Differences in trichothecene structure have been studied in order to determine the effects that substituent groups of the backbone may have on animal and plant toxicosis. Studies based on natural and synthetic trichothecenes have identified structural features which convey higher or lower toxicity in plant and animal systems [75,82,83,85,92]. For example, Anderson *et al*. [92], tested forty-two compounds, derived from modifications of T-2 toxin or neosolaniolagainst mouse lymphoma cells for antileukemic properties. It was observed that the cytotoxicity was altered by modifications at C-3, C-4, C-9 and C-10 [92]. By contrast, changes at C-8 resulted in minimal changes in cytotoxicity, indicating a region of steric tolerance [92]. While it is evident that differences in the R-groups can affect cytoxicity, the structural and mechanistic reasons for these differences are unknown.

In order to gain a better understanding of the toxicological properties of trichothecenes and other toxins, it is important to understand the structural dynamics of these compounds and how they might interact with the cellular environment. To this end, new and more advanced methods must be used for an adequate treatment of these compounds. We are of the opinion that extensive solution, solid-state and NMR crystallographic studies in conjunction with molecular dynamics calculations of trichothecene structure, dynamics and molecular interactions are essential methods to determining the mode of action of trichothecenes.

## 3. Methods to Study Structure

When the trichothecene, trichothecin, was isolated by Freeman and Morrison in 1948 [18], the sophisticated techniques used to study small molecules today were not available. The structure of this compound was not described until 1959 when the Freeman group used a form of fingerprinting, or chemical modification, to determine the toxin core and substituent groups [98]. Although a valid technique, chemical modification is time-consuming and does not provide the absolute stereochemistry of a compound. Furthermore, any information regarding the flexibility, electronics, or three-dimensional configuration of the molecule is lost. Consequently, as new techniques in structure determination and fungal fermentation were made available, the number of trichothecenes isolated and identified began to grow dramatically [9]. Although, Mass Spectrometry and Fourier-transform Infrared Spectroscopy can offer some insight into the chemical structure of organic molecules [99,100,101], they do not provide a complete picture on their own and merely serve as pieces to the puzzle; therefore, they will not be considered further in this review. Of particular interest to the complete structural identification of trichothecenes is the information made available from X-ray Crystallography and Nuclear Magnetic Resonance (NMR), which will be dealt with in detail here.

### 3.1. Crystallography

X-ray Crystallography is unrivalled in the structural detail and accuracy it can provide, and is thus highly valued in virtually all branches of chemistry. Unfortunately there are many limitations imposed on its application (some of which will be described below), and as such it has not been able to truly become a routine characterization method readily accessible to all chemists. Recent advances in instrumentation have alleviated some of these restrictions and thus it is seeing much wider application to inorganic, organic, biological, and some aspects of materials sciences [102,103].

The X-ray Crystallography experiment uses high intensity x-ray beams, which are focused on the crystal. The X-ray irradiation is scattered by the crystal in many specific directions. The resultant diffraction pattern is defined by the electron density, and corresponds to the regularly spaced arrays of atoms within the crystal [104]. For each orientation of the crystal with respect to the incident beam there is a corresponding diffraction pattern. A series of two-dimensional diffraction patterns are collected over as many orientations as is practicable, and are combined to create a three-dimensional working structural model of the density of electrons within the crystal. Diffraction data are collected in reciprocal space and must be converted to Cartesian co-ordinates in order to be analysed; the data conversion is achieved through the use of the mathematical method of Fourier Transformation. This model is compared with chemical information known from experiment, and can then be refined and optimized to obtain the best fitting structure. The structural model is composed of the lattice structure, which is determined by the arrangement of the smallest building block, known as the unit cell, and the arrangement of the molecules therein in their respective configurations [103]. 

The quality of the diffraction pattern obtained is thus highly dependent on the quality of the crystal used and the degree to which the electron density is able to scatter the radiation [103]. The latter is simply a matter of the number of electrons present around the atomic species in the lattice; hence, the higher the atomic weight, the better the data. The former has historically been the major obstacle. The crystals have to be as large as possible to give the best quality diffraction data; however, it also must be free from defects. The crystal must have long range order, with no deviation from the regular pattern due to the molecular structure and unit cell. 

Thus, the best quality diffraction data are those which can be obtained from Single Crystal X-ray Diffraction (SCXRD) analysis. However, there are many sources of disorder inherent to the crystallization process, including: the coexistence of various crystalline forms, or polymorphism [103]; the inclusion of additional species within the crystal, such as solvent [103]; and motion, such as the libration of some portion of the molecule or in some cases the reorientation of the entire molecule [104]. Often single crystals of sufficient size cannot be achieved, in which case diffraction measurements can be pursued on powders. Unit cell dimensions can be obtained from powders, but does not provide as much structural information as can be obtained from crystals since, for powder diffraction, the three-dimensional data must be projected onto a one-dimensional plane in order to map the diffraction pattern [104]. Structural parameters regarding the dimensions of the unit cell are invaluable when phase information is of interest, such as for semi-crystalline, polymeric, and ceramic materials. 

Historically speaking, SCXRD has seen extensive use by inorganic chemists, but as the instruments and crystallization methods improved, organic and biological systems were increasingly investigated. The latter two of course suffer from being composed almost entirely of relatively low atomic weight species, and pose more challenges in obtaining crystals of sufficient quality and size. Furthermore, in organic and biological systems the position of hydrogen atoms is often extremely important to relating structure and function, as is the case with hydrogen bonding, and thus can limit the interest in the technique. With the emergence of high intensity X-ray sources, and very sensitive array detection, SCXRD of biological macromolecules has become largely a matter of making crystals [103]. However, the question still remains as to how the crystal structure relates to the actual biologically active configuration. These are relatively recent advances, and as a result, SCXRD did not make a large contribution to the early literature in structural studies of natural products such as trichothecenes. Today’s experimental capabilities do offer a tremendous opportunity to expand the structural understanding of these systems, especially regarding the interaction of trichothecenes with biological systems, such as proteins, DNA and RNA [103]. 

The main contribution of SCXRD to trichothecene literature has been the determination of the absolute stereochemistry of the side chains in the macrocyclic systems. One of the earliest studies was performed on verrucarin A from *Myrothecium verrucaria*, in 1966 by McPhail *et al*. [105]. In this study, phasing of the diffraction pattern was problematic, and verrucarin A had to be co-crystallized with p-iodobenzensulfonate; thereby, incorporating strongly scattering Iodine and Sulfur atoms to help with the phase determination. It was determined that the absolute configuration of the rings of the trichothecene backbone for verrucarin A were in a half-chair for the A-ring, while the B-ring was in a chair conformation, and the C-ring was in an envelope configuration (Figure 2A). The side chain was confirmed to link C-4 and C-15 and the absolute stereochemical configurations of two of the side-chain carbons, C-2′ (S) and C-3′ (R), were determined. A later study by Soriano-Garcia *et al*. in 1999 [106], provided a high resolution structure of the pure compound, confirming the observations by McPhail *et al*. [105], and providing details on an intermolecular hydrogen-bonding occurring between the hydroxyl group on C-2′ and the carbonyl oxygen on C-1′. Related work by Anderson in 1988 [107], also confirmed the McPhail structure for verrucarin A, and compared it with a series of analogous artificial macrocyclic systems. The macrocycle was likened with chiral crown ethers, and their results revealed that the natural systems had a strong binding preference for RNH_4_^+^ functionalities with R = H, CH_3_, and t-butyl. It was proposed that selective binding of this type could occur between -NH_3_^+^ sites on proteins associated with the 60S ribosome. The structure for verrucarin B was determined by Breitenstein in 1979 [108], which has an epoxide functionality at the C-2′ and C-3′ position. The absolute configuration at C-2′ and C3′ was shown to be *S* and *R*, respectively, as was observed by McPhail for verrucarin A. SCXRD also played in important role in the structures of the roridins particularly in distinguishing between roridin A and isororidin A. These macrocycles are related to the verrucarins, where the ester linkage at C-6′ is transformed to an ether, with a hydroxyl ethyl functionality attached at C-13′. The two forms are distinguished by the stereochemistry about the C-13′. Due to the lack of chemical shift dispersion for ^1^H and ^13^C, the NMR spectra of the two compounds were nearly identical [109]. In 1982, Jarvis *et al*. employed SCXRD to verify the configurations about C-13′ [109], which was found to be *R* for roridin A and *S* for isororidin A. Later, in 1987, Jarvis made use of SCXRD in a similar way for the baccharinoids B1, B2, B3, B7 [30]. First, it was determined that the baccharinoids B3 and B7 were epimeric at C-13′, similar to what was observed for roridin A and isororidin; furthermore, B7 was seen to be epimeric with Roridin A at C-2′. Again ^1^H and ^13^C NMR were not able to convincingly distinguish between the two configurations. Jarvis *et al*. also crystallized the triacetate of B2, and found that B1 and B2 were also epimeric at C-13′. In 1999, the same group was able to employ insights from these X-ray studies in an investigation into the diastereomers of roridin E [110], following the work by Flippen-Anderson in 1986 [111], in order to infer the absolute configuration of various carbons in the sidechain, on the basis of ^13^C NMR chemical shift differences near the stereogenic centers. The latest SCXRD work on the roridins was published by Gai in 2007 [112]. Here, the analysis of roridin H again focused on the stereochemistry of the sidechain, this time at positions C-4′, C-5′ and C-6′, which are part of a five-membered ring. 

Jarvis *et al*. also investigated another class of macrocycles known as the myrotoxins isolated from *Myrothecium roridum* [113]. These systems include a tetrahydropyranyl ring, fused to an epoxide, in the side chain from C-2′ to C-6′. In this case, the absolute configuration about C-2′, C-3′, and C-6′, were essential in order to properly describe the stereochemistry of the rings in the sidechain. The orientation of the hydroxyl at C-12′ was used to distinguish between the A and C isoforms, of which the latter is significantly less stable. A recent work by Shen *et al*. in 2006 [5], looked into the structures of a series of related macrocycles produced by *M. roridum*, myrothecenes, that also contain a tetrahydropyranyl function. High resolution SCXRD structures of myrothecene A and C were obtained to gain insight into the stereochemistry of the tetrahydropyranyl, which was in the chair form in both cases, where the absolute configuration about C-6′ and C-12′ were shown to be essential to the assignment. It was also found that there was insufficient space in the ring of form A to encapsulate water, while in form C, a water molecule was incorporated, and found to be involved in a hydrogen bonding interaction with O-5, O-3, and O-2 [5]. 

Type A and B trichothecenes have had comparatively little X-ray Crystallography analysis, presumably since less stereochemical variation would be expected in the trichothecene backbone as compared with the sidechain of the macrocyclic systems. The structure of DON was attempted on several occasions [114,115]; however, crystals of sufficient quality could not be obtained. In contrast, suitable crystals for 3-*O-*acetyldeoxynivalenol (3-ADON) were acquired in 1984 by Greenhalgh *et al*. [114], and a high resolution structure was obtained. It was determined that the absolute configurations of the backbone carbons were essentially identical to those found in verrucarin A by McPhail *et al*. [105]. The unit cell for 3-ADON is composed of layers of four molecules arranged in tetrameric rings along a 4-fold rotation axis. The centers of the tetramers form channels large enough to include solvent, which accounted for the different crystal forms observed when 3-ADON is recrystallized from different solvents. A significant amount of disordered water molecules were shown to be included in the crystals in the aforementioned channels. In a second failed attempt in obtaining DON crystals suitable for SCXRD, Greenhalgh and coworkers were able to discern that at least two forms of DON exist, which differ in the amount of water included [115].

In 1990, Gilardi *et al*. performed an SCXRD study on T-2 Toxin [116], and the stereochemistry of the rings were found to be identical to that of verrucarin A [105]. The unit cell for T-2 toxin, contains two distinct molecules differing in the configuration of the isovalerate sidechain. The relatively large thermal-ellipsoid employed for the atoms in the sidechains as opposed to those in the core, testify to a significant degree of disorder [116]. Also, hydrogen bonding was observed involving O-1 and O-2, which were shown to donate into the hydroxyl hydrogen at C-7 [116]. 

**Figure 4:**
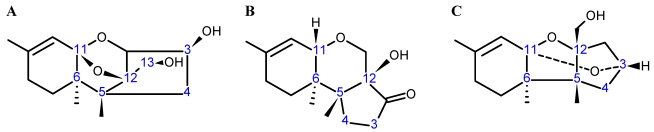
Structures of some non-trichothecene trichodienoid compounds: (**A**) sambucinol; (**B**) sambucoin; (**C**) 13-hydroxy-3α,11-epoxyapotrichothecene.

The X-ray Crystallographic information available in the trichothecene literature is quite sparse; however, many studies on the structure of related trichodienoid compounds exist. For example, the structure of trichodienoids sambucinol and sambucoin, isolated from *Fusarium sambucinum*, were investigated by Mohr *et al*. in 1984 [117]. The trans-ring fusion between the A and B rings was shown to form an oxygen bridge from C-11 to C-12 in sambucinol (Figure 4A). For both derivatives, the absolute configuration of the ring and the loss of the epoxide function was confirmed by the X-ray data (Figure 4A,B), and of particular note was the retention of the *S* configuration about C-12 [117]. In a later study of secondary metabolites of *Fusarium sporotrichioides*, Greenhalgh *et al*. observed a molecule, 13-hydroxy-3α,11-epoxyapotrichothecene (Figure 4C), where the epoxy ring is opened and the 3-OH is lost to form an oxygen bridge from C-3 and C-11 [34]. Although, many of their observations were made by NMR, the absolute configuration was confirmed by the SCXRD.

X-ray Crystallographic analysis can also be applied to trichothecenes in complex with interacting proteins. Garvey *et al.* studied the structure and function of the trichothecene 3-*O*-acetyltransferase (TRI101) from *F. graminearum*, and trichothecene 15-*O*-acetyltransferase protein (TRI3) from *F. sporotrichioides*, in 2008 and 2009, respectively [55,56]. TRI101 was complexed with CoA and DON together, as well as with T-2 toxin and CoA [55], and TRI3 in complex with decalonectrin and DON [56], were performed. Although these studies focused primarily on the X-ray crystal structure of the protein, important information regarding the trichothecene binding site for both TRI101 and TRI3 were unambiguously identified. The determination of the structural dynamics of the trichothecene class of toxins becomes important when considering how such binding interactions occur.

### 3.2. Nuclear Magnetic Resonance (NMR)

Structure determination of the majority of organic, biological and inorganic molecules often begins with Nuclear Magnetic Resonance (NMR) spectroscopy. Aside from crystallography, which possesses the ability to discern the complete molecular structure of pure crystalline materials, NMR is the most direct tool for identifying the structure of not only pure compounds, but can also be helpful in the determination of mixtures of compounds in both the liquid and solid-state. Historically, NMR lacked the ability to give precise geometrical information, such as bond lengths and angles. However, it is able to identify chemical environments and their interconnectivities from which the structure can be inferred. As such, absolute configurations cannot be determined by NMR. Information regarding configuration can only be proposed relative to some initial position of reference. 

NMR is a spectroscopic technique which exploits the intrinsic magnetic properties of atomic nuclei. When a strong external magnetic field is applied, nuclei undergo precessional motion (similar to that observed when a child spins a top) at a frequency, ω_o_, known as the Larmor frequency. The Larmor frequency is defined by the strength of the external magnetic field and the gyromagnetic ratio of the nucleus [118]. The gyromagnetic ratio, γ, is a constant which is unique for each type of nucleus; therefore, each type of nucleus has its own characteristic Larmor frequency. Subtle differences in the electronic environment surrounding each individual atom in a molecule lead to a distortion of the magnetic field experienced by the nucleus. This effect is known as shielding, and results in a distinct frequency shift, ν_o_, or chemical shift, for each chemical environment of the nucleus [119]. Distortions in the field also arise due to the magnetic moments of nearby nuclei, and result in a splitting of the signal known as coupling. Coupling may occur either through-space (direct spin-spin coupling) or through-bond (indirect spin-spin coupling) [120]. In solution-state NMR experiments, through-bond coupling is observed, while the through-space interactions are removed due to random reorientations of the molecule [121]. The strength of the coupling interaction, known as the coupling constant, J, is a function of the electron density between the nuclei in question; therefore, the coupling is highly indicative of the intervening structure [120]. Scalar coupling is typically observed for nuclei which are 2 (geminal) or 3 (vicinal) bonds apart from each other; however, longer range coupling may be observed for rigid or π-bonded systems [122]. Vicinal coupling constants can provide useful conformational information through calculations involving the Karplus equation. The through-bond coupling interaction allows for the molecule to be stitched together bond by bond, providing an overall picture of the connectivity of the molecule. 

NMR spectroscopy was originally developed in two different labs [119], those of Bloch and Purcell, in 1946. However, the technology did not become commercially available until the late 1950s. At this time, the technique was still in its infancy, and spectra were available only for very sensitive nuclei at magnetic fields with ^1^H frequencies around 60 to 100 MHz. In the 1970s, superconducting magnets for NMR were made available, offering higher field strength for the separation of signals and providing an opportunity for the study of lower sensitivity nuclei, such as ^13^C. Today, NMR spectrometers with magnets of 23.5 T are available, which corresponds to a ^1^H resonance of 1 GHz.

The major breakthrough in NMR spectroscopy remains the advent of Fourier Transform NMR (FT-NMR) [123]. Fourier transformation is the mathematical conversion of the NMR signal from a function of time(s) to a function of frequency (s^−1^ or Hz). The development of Fourier theory ultimately led to the development of multi-dimensional NMR techniques, which allowed for the study of more complex molecular systems, including proteins and nucleic acids.

Multi-dimensional NMR analysis of organic compounds and natural products comes in a variety of different forms, based on the type of nuclei being correlated and the interaction on which the correlation is established [118]. Homonuclear correlation experiments correlate two nuclei of the same type. Correlation Spectroscopy (COSY) is based on the direct coupling interaction typically over two to three bonds; whereas, Total Correlation Spectroscopy (TOCSY) correlates all nuclei which are part of a coupled network, either directly coupled, or where the coupling is relayed through some mutually coupled partner. Nuclear Overhauser Effect Spectroscopy (NOESY) correlates nuclei through space via the direct dipolar coupling interaction, identifying other proximal nuclei [124]. Multi-dimensional NMR spectroscopies have evolved to a high level of sophistication making correlations over 3 to 4 bonds, and are routinely used to study large macromolecules including proteins, DNA and RNA, not only *in vitro* but also *in vivo*, through specialized in-cell NMR techniques [125,126]. 

NMR is not a sensitive technique as the signal arises only from isotopes that are NMR active and is related to its Larmor frequency. Thus, rare nuclei with low frequencies such as carbon are insensitive, in comparison to hydrogen, and may require polarization transfer methods to enhance their signal, or by employing isotopic enrichment of a sample. Polarization transfer exploits the coupling between hydrogen and carbon atoms, to transfer the much larger proton polarization to the carbon nucleus, through the irradiation of the ^1^H resonance, or by employing a specialized pulse sequence. The signal is thereby enhanced by a factor related to the ratio of the ^1^H and ^13^C gyromagnetic ratios. The recent development of the Dynamic Nuclear Polarization (DNP) technique is certain to revolutionize structural identification through NMR [127]. DNP expands on the idea of polarization transfer to unpaired electrons, where the electron polarization of a radical is transferred to nearby nuclei by irradiating the electron resonance using a microwave source. As the gyromagnetic ratio of an unpaired electron is several orders of magnitude greater than that for ^1^H, the signal enhancement achieved through polarization transfer from an electron is quite dramatic. For example, a 400 MHz NMR magnet with DNP-enhancement is capable of delivering microwaves up to 263 GHz [127], and can receive a signal-to-noise enhancement up to 80 times that typical for the nuclei observed.

### 3.3. Solution NMR and the Structure Determination of Trichothecenes

NMR is the principal technique used in the determination of the structure and stereochemistry of trichothecenes, with over 90% of trichothecenes being characterized in this manner. Extensive reviews regarding the characterization of trichothecenes and trichothecene-related compounds have been published [24,25,27,30,35], and the reader is directed to these works for more information regarding the structural parameters of these compounds.

Early NMR characterization was limited to relatively low field ^1^H NMR. The early compilation of trichothecene chemical data in Cole and Cox in 1981 [1] featured 100 MHz or less NMR spectra which provided no coupling information. ^13^C assignments were obtained at 25 MHz and employed off-resonance decoupling [128], leading to disagreements in the literature regarding assignments in the 1970’s. With better knowledge of the production and purification of trichothecenes in the 1980s, pure standards could be produced in sufficient quantity for complete characterization. With availability of 2D experiments, specifically homonuclear (^1^H-^1^H) and heteronuclear (^1^H-^13^C) direct correlation spectroscopies, even poorly resolved resonances could be accurately assigned, leading to the correct assignment of the ^1^H and ^13^C skeleton of several key trichothecenes [129]. As field strengths increased and longer range 2D experiments were made available, definitive assignments of the quaternary carbons were made. These accurate assignments were used for biosynthetic studies and to design labeled material for toxicological studies in animals. As more trichothecenes were isolated and characterized, trends were observed in the NMR data permitting the assignments of unknown structures on the ^1^H NMR alone [24], these trends are described below. The NMR information provided in Savard and Blackwell [25], for more than 50 trichothecenes and related compounds from *Fusarium* species permit the identification of new metabolites as they arise. 

Trichothecene mycotoxins display some rather prominent structural features [24]. The most recognizable of which is the methylene ^1^H couplings of the epoxide ring found in the window of 2.7 to 3.4 ppm, with a J-coupling of approximately 4 Hz. However, for some of the derivatized trichothecenes, the coupling of this AB system collapses to a singlet. The methyl hydrogens at C-14 appear as a sharp singlet, whereas those at C-16 appear broad due to long range coupling with H-10. Long range coupling from H-7β to H-11 can be observed due to the “W” relationship between them, indicating a very rigid ring system, and necessitating the half-chair and chair conformations for the A-ring and B-ring, respectively [24] (see Figure 2). 

NMR spectroscopy of trichothecenes is limited primarily to ^1^H and ^13^C methods. Basic one-dimensional ^1^H NMR experiments of natural products the size of trichothecenes at today’s magnetic field strengths (300 to 900 MHz) are often sufficient to determine the number and type of ^1^H environments, and their connectivity from the chemical shifts and coupling patterns of the signals. The fact that such high quality structures were obtained of these systems in 1960’s and 1970’s, before two-dimensional NMR methods became available, was no minor feat. 

**Figure 5:**
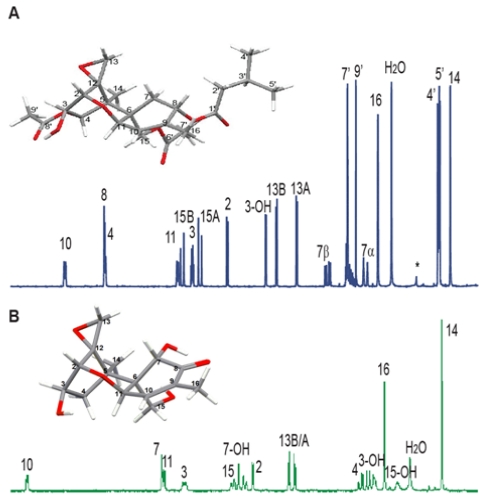
The ^1^H NMR spectra of (**A**) T-2 toxin and (**B**) Deoxynivalenol (DON) at 300 MHz in CDCl_3_. All ^1^H resonances have been assigned and are labeled.

The ^1^H spectra for T-2 toxin and DON, shown in Figure 5, were taken at 300 MHz [128]. All the resonances of the backbone structure can be identified; only the resonances of the isovalerate side chain at C-8 and acetyl functions at C-4 and C-15 of T-2 toxin were difficult to assign. The methyl resonances, H-14 and H-16, were identified by their low chemical shift and lack of splitting, plus long range coupling of H-16 to H-10. The methyl resonance of the acetate function at C-4 occurs at higher chemical shift than that of C-15, and those on the isovalerate function (H-4', H-5') are doublets due to a vicinal coupling with their neighboring methine proton, 3'.

**Figure 6:**
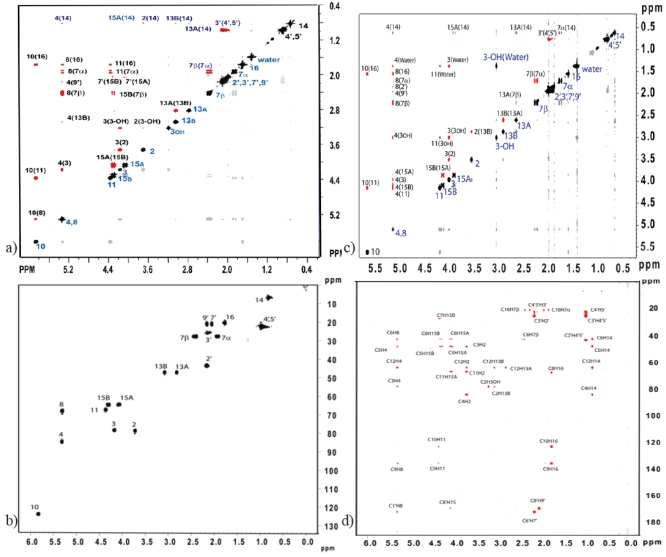
Two-dimensional NMR spectra of T-2 toxin. (**A**) Correlation Spectroscopy (COSY) spectrum showing ^1^H-^1^H homonuclear through-bond (J-coupling) correlations; (**B**) Nuclear Overhauser Effect Spectroscopy (NOESY) spectrum depicting ^1^H-^1^H homonuclear through-space (dipolar coupling) correlations; (**C**) Heteronuclear Single Quantum Correlation (HSQC) spectroscopy spectrum depicting ^1^H-^13^C correlations for carbon nuclei which are directly bound to one or more ^1^H nuclei; (**D**) Heteronuclear Multiple Bond Correlation (HMBC) spectroscopy spectrum depicting ^1^H-^13^C correlations for carbon nuclei which are up to 4 bonds away from one or more ^1^H nuclei.

Trichothecenes have a double bond in the A-ring, which increases the chemical shift of nearby protons, H-16 and H-10. The methine proton H-10 undergoes a vicinal coupling with H-11, and has a longer range coupling to H-8. H-8 has vicinal couplings to both protons on C-7. Crosspeaks in the COSY spectrum support the proton assignments of ring A (see Figure 6A). The protons of the epoxide ring, H-13A and H-13B are readily identified by their chemical shift and AB pattern with a characteristically small geminal coupling indicative of high ring strain, which also has a corresponding cross peak in the COSY spectrum. The remaining protons on the C-ring can be assigned by first identifying H-3OH by its ability to be replaced by a deuterium upon addition of D2O. H-3OH has a vicinal coupling to H-3, which in turn has vicinal couplings to H-2 and H-4. The NOESY spectrum, shown in Figure 6B can be used to distinguish between the hydrogens in different stereochemical positions on C-7, C-13 and C-15. 

The carbon resonances can be assigned on the basis of their chemical shifts and employing multiplicity selection sequences such as Distortionless Enhancement by Polarization Transfer (DEPT), where the signal phase indicates the number of attached protons [130]. Their assignment can be confirmed using Heteronuclear Single Quantum Correlation (HSQC) and Heteronuclear Multiple Bond Coherence (HMBC) correlations, see Figure 6C,D, respectively [119]. In order to determine the relative structural conformation of the trichothecene core, Savard *et al*. conducted an extensive ^1^H NMR study on 36 natural and synthetic trichothecene compounds, based on the Type A and B trichothecenes [25]. The major conclusion from this study was the finding that the most stable conformation of the trichothecene core occurs when the A-ring assumes a half-chair conformation, and the B-ring a chair conformation. 

**Figure 7:**
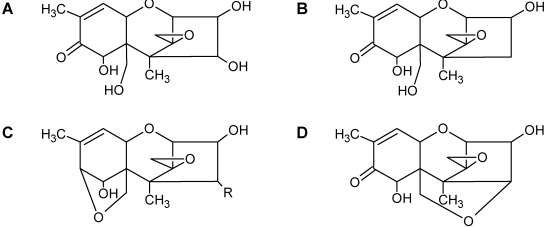
Structural changes observed for NIV and DON under different solvent conditions: (**A**) chemical structure of NIV; (**B**) chemical structure of DON; (**C**) hemiketal formation from C-8 to C-15 observed for NIV and DON, where R is -OH and -H for NIV and DON, respectively; (**D**) cyclic ether formation from C-4 to C-15 observed for NIV only.

The NMR spectroscopy performed on trichothecenes has been done primarily in solution. Solution-state NMR offers the ability to study compounds in a variety of solvents, under various conditions (*i.e.*, pH, polarity, dielectric constants), which may influence the chemical nature of the compound. Furthermore, studies can be performed under physiological conditions, at various concentrations. However, the majority of NMR studies performed on trichothecenes have been done in deuterated chloroform (CDCl_3_), a mildly polar solvent, with a polarity index of 4.1 as compared to water which has a polarity of 9.0; furthermore, CDCl_3_ lacks the ability to undergo hydrogen bonding. Although CDCl_3_ is a principal solvent for NMR studies for the purpose of structural determination, it is possible for compounds to adopt alternative conformations when observed under different solvent systems. In a study by Jarvis *et al*. in 1990 [26], different solvent systems were used to determine the conformational effects of solvents on the trichothecenes NIV and DON [26]. DON was observed in DMSO-d_6_, (CD_3_)_2_CO and CD_3_OD, whereas NIV was only studied in DMSO-d_6_ due to insolubility in the other solvents. NIV and DON are Type B trichothecenes, characterized by the presence of a ketone functionality at C-8; however, it was determined that under different solvent systems, it is possible for this position to adopt a hemiketal functionality, where an ether linkage is formed between C-8 and C-15 [26]. Jarvis determined that in DMSO-d_6_, DON is present in the hemiketal form in approximately 10% of the relative population, and 6% in (CD_3_)_2_CO. No evidence of the hemiketal configuration was observed in CD_3_OD or CDCl_3_. NIV differs from DON only in the substituent group at C-4; NIV has a hydroxyl group at this position, whereas DON is non-substituted. Thus, NIV also has the ability to form a cyclic ether from C-4 to C-15 [26], similar in many respects to the linkage observed for Type D macrocyclic trichothecenes, see Figure 7. Under these conditions, the tetrahydropyranyl B-ring, which typically has a strong preference for a chair configuration, is observed to adopt a boat configuration [26].

A similar observation has been made for the antibiotic, virginiamycin 1 (VM1) by Dang *et al*. in 2004 [131]. In an NMR analysis of the antibiotic, it was shown that VM1 adopts a different conformation when studied in CDCl_3_, from what is observed when it is bound to the 50S bacterial ribosome [132]. High resolution two-dimensional NMR spectra were obtained for VM1 in DMSO-d_6_ and CD_3_OD which not only differ from the three-dimensional structure of VM1 in CDCl_3_, but also from the bound structure.

These observations regarding alternative conformations of trichothecene toxins, and other related compounds, in different solvent systems are important when considering the conformation which these toxins may adopt under physiological conditions. However, to the best of our knowledge, only one study, that performed by Duffy and Reid on the stability of T-2 toxin in aqueous solution, has been performed on trichothecenes under quasi-physiological conditions at this point [133]. This is likely due to solubility issues at the concentrations required to obtain atomic resolution structural information by solution-state NMR. The study on T-2 toxin, however, was not strictly a structural investigation, but rather focused primarily on the stability of T-2 toxin in phosphate-buffered solutions in the pH range of 5-12 over the course of one year. It was determined that T-2 toxin is extremely stable under pseudophysiological conditions, with the epoxide functionality remaining remarkably intact after 4 years in solution, indicating that the detoxification of T-2 toxin is most likely an enzymatic process [133]. The stability of the epoxide ring in solution is rather astounding considering the substantial amount of ring strain inherent in the three-membered cycle [133,134,135].

**Figure 8:**
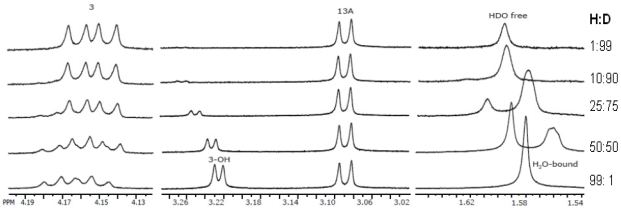
Deuterium exchange experiment performed on T-2 toxin. Regions exhibiting significant changes throughout the incremental addition of D_2_O have been expanded to show peak structure. Of particular interest are the H_3OH_ and H_2_O/HDO regions, which not only demonstrate significant changes in chemical shift, but also exhibit the retention of their sharp peak structure, indicating a slow chemical exchange process. H_3_ is less affected in that the only observable changes are the loss of coupling to H_3OH_ as the latter peak is converted to H_3OD_.

Deuterium exchange and variable temperature experiments are useful tools in the assignment of exchangeable ^1^H resonances, such as hydroxyl, amine, amide and sulfhydryl bonds [136]. Although, exchanging ^1^H resonances often appear as broad lines in the ^1^H spectrum, hydrogen-bonding interactions can have a significant effect on the rate of exchange of a ^1^H, resulting in a line-width of the same order as the other peaks in the spectrum. If the rate of exchange, *k*_ex_, is longer than the time it takes for the system to relax, T_1_, no line broadening due to exchange will be observed [136]. By adding deuterated water, D_2_O, to the sample, signals resulting from exchangeable ^1^Hs will effectively disappear from the spectrum, as the ^1^Hs are replaced by ^2^H nuclei, which appear at a different spectral frequency than ^1^H. Furthermore, if scalar coupling to an exchangeable ^1^H is observed, that coupling will also disappear in the resonances to which it is coupled, due to the incorporation of ^2^H. Intermolecular interactions of a compound with water can also be confirmed through the analysis of a deuterium exchange experiment. In an experiment performed by our lab on T-2 toxin [57], couplings to water were observed in the two-dimensional NOESY spectrum, to resonances in the same vicinity as the exchangeable hydroxyl bound to C-3 at position R_1_, surrounding the tetrahydropyranyl pocket which forms as a result of the three-dimensional configuration of the trichothecene. In order to confirm that water was indeed bound within this pocket, D_2_O was added dropwise to the NMR sample tube. H_2_O was found to be in slow exchange with D_2_O, suggesting that at least one water molecule is tightly bound to this region of the molecule (see Figure 8) [57]. Furthermore, the temperature dependence of hydroxyl proton peaks can often be used to assign them, where variable temperature NMR is a possibility.

Structural parameters obtained through NMR can be employed directly in molecular modeling studies to help in describing their lowest energy conformations and how they change over time and interact with their surroundings [104]. This type of molecular modeling is useful for gaining insight into mechanistic behavior, and predicting the structure of likely intermediates. At this point, very few molecular modeling and dynamics studies have been performed on the trichothecene class of mycotoxins; however, those that have been attempted have used solution-state NMR spectral parameters to model the system. Of note are the studies performed on DON by Nagy *et al.* [xref^137^], verrucarin A by Fragaki *et al*., and both verrucarin A and roridin A by Steinmetz *et al*. [138,139]. In all cases, molecular dynamics were employed to determine the relative conformations of the toxins in question, in order to better understand whether a single conformation is predominant, or whether multiple conformations exist simultaneously under physiological conditions. MD simulations of these compounds were able to determine that in all likelihood DON exists in a single conformation which is entropically favored due to intramolecular hydrogen bonding interactions [137]. Verrucarin A predominantly exists in a single conformation which accounts for approximately 75% of the total relative population, while several other low-energy conformers account for the remaining population [138]; whereas, roridin A exists as a mixture of two nearly equi-energetic conformers [139].

### 3.4. Spectroscopic Determination of Trichothecenes Through Solid-State NMR

**Figure 9:**
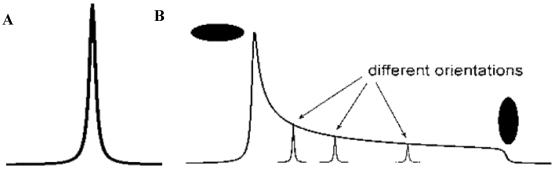
Appearance of NMR signals under (**A**) isotropic, and (**B**) anistropic experimental conditions. Samples rotationally averaged by the Brownian motion of a solvent in the solution-state; or solid-state samples which are rotationally averaged due to Magic-Angle Spinning (MAS), will have NMR lineshapes similar to that depicted in (**A**). Solid-state samples experiencing multiple orientations simultaneously will display powder patterns similar to that depicted in (**B**).

It is also important to note that NMR structural studies are not limited to the solution-state. Until recently, solid-state NMR has been plagued with difficulties in spectral resolution due to the extreme line broadening caused by the orientational dependence of shielding and coupling interactions. However, advancements in high-field spectrometers, ultrafast sample spinning, and new pulse sequence techniques, have made the study of organic compounds via solid-state NMR a reality. In solution, molecules rotate rapidly in random orientations averaging the signal isotropically [119]. In contrast, molecules in the solid-state are essentially static; thus, in a powder, the entire range of orientation is manifested as a broadened signal, covering a very large range in frequency, with an intensity pattern reflecting the relative probability of each orientation. This is referred to as a powder pattern (see Figure 9).

The anisotropy of the shielding and coupling interactions can be removed, to a large extent, by spinning the sample at an angle that corresponds to the body diagonal of a cube, obeying the rule (3cos^2^Θ − 1) = 0. The angle Θ, equal to 54.74°, is referred to as the magic angle. Magic Angle Spinning (MAS) of a solid powder mimics the isotropic motion seen in solution, and leads to averaging of the NMR signal to a single frequency if the sample is spun at sufficient speed; otherwise a side band pattern is observed [140]. 

Spinning of the sample also has the potential to remove other interactions that are present in the solid-state that are not seen in solution, as motional averaging effectively removes them. Dipolar coupling is observed in the solid-state and arises due to the direct interaction between the magnetic moment of one spin sensing the magnetic field resulting from the magnetic moment of another spin. The magnitude of this coupling effect is proportional to (γ*_i_*γ*_j_*/*r*^3^); where γ*_i_* is the gyromagnetic ratio of the first spin, γ*_j_* is the gyromagnetic ratio of the second spin, and r is the internuclear distance [141]; thus, it is possible to determine the distance between two dipolar coupled nuclei, using this relationship. Coupling between two like nuclei, homonuclear coupling, gives rise to line-broadening. If the rate of sample spinning is greater than the magnitude of this interaction, the coupling is effectively removed and with it any consequent line-broadening. Conversely, if the dipolar coupling exceeds the spinning speed, the contribution to the line width can be significant. The latter situation occurs between ^1^H resonances, producing broad and featureless spectra which are of little utility on their own.

Recent advances in the field of solid-state NMR has led to the introduction of new techniques and pulse sequences which have the ability to navigate around the issues of large chemical shift anisotropies and strong dipolar coupling. Ultrafast MAS probes have been designed that can spin up to 80 kHz, in an attempt to effectively suppress dipolar coupling interactions between strongly coupled nuclei, such as ^1^H homonuclear coupling. Furthermore, pulse sequences, such as Phase-Modulated Lee Goldberg (PMLG) decoupling, can be used to average out the zero- and first-order dipolar coupling terms in the Hamiltonian and effectively mimic the effects of ultrafast sample spinning at more moderate spinning speeds [142]. 

The importance of studying biologically active compounds in the solid-state has been illustrated in a review by Geppi *et al*., regarding the solid-state NMR analysis of pharmaceuticals [143]. Of particular importance is the high relative occurrence of polymorphism among biological and organic compounds. It has been demonstrated that in many cases, the biological activity between polymorphs varies, even though they all share the same chemical structure and stereochemistry. It is often extremely difficult to obtain individual single crystals of polymorphic compounds, since there is a high degree of disorder, due to the different conformations observed. Given the evidence for the inefficiency of crystallization of trichothecenes [115], and the different conformations observed due to solvent properties [26], it is likely that different polymorphs of these compounds may be present. Therefore, it is the impression of the authors that studying the compounds in the solid-state may prove to be beneficial to understanding the overall structure-function relationship and general toxicology of these compounds.

**Figure 10:**
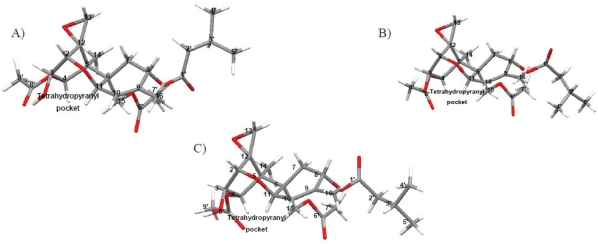
Solution- and solid-state NMR structures observed for T-2 toxin: (**A**) solution-state conformation of T-2 toxin observed in CDCl_3_; (**B** and **C**) two different solid-state conformations for T-2 toxin, differing mainly in the orientation of the side chains.

**Figure 11:**
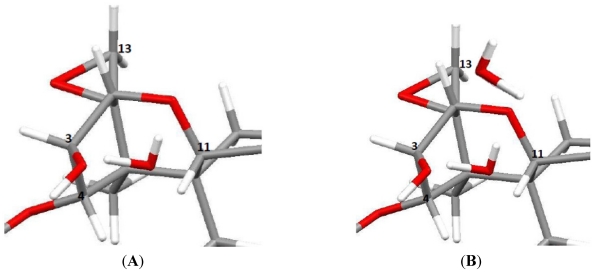
Proposed water binding interaction for T-2 toxin in the (**A**) solution- and (**B**) solid-state. At least one molecule of water has been observed to bind in the pocket formed between the tetrahydropyranyl B-ring and cyclopentyl C-ring. The major chemical shift differences observed for T-2 toxin in the solid-state NMR spectrum suggest that a second water binding site may be present near C-12 of the epoxide ring.

To the best of our knowledge, the only solid-state NMR study performed on trichothecene toxins to date has been contributed by our lab [57]. In this study, it was determined that T-2 toxin adopts three distinct conformations: one in solution, and two different conformations in the solid-state, shown in Figure 10. In previous studies, water had been classified as a contaminant, and even after vacuum pumping on a sample for several hours, the water peak remained [144]. However, it is our impression that water is not necessarily a contaminant, but rather is a key feature of the trichothecene structure. Water bridging is an important feature of many biological compounds, often resulting in a rigidification of the structure in order to confer activity [145]. In solution, a water molecule was observed to sit in the “pocket” created by the tetrahydropyranyl B-ring and cyclopentyl C-ring, rigidifying the structure, as evidenced by our deuterium exchange and NOESY experiments [57]. In the solid-state, a second weaker water binding is believed to exist on the opposite side of the molecule near the epoxide [57], which is evidenced by the large chemical shift differences observed in the solid-state for these particular signals, the proposed interactions with water are depicted in Figure 11.

Solid-state NMR, although a highly effective technique on its own, has recently been shown to be far more effective when combined with X-ray diffraction studies and molecular dynamics, to provide atomic resolution structures of complex systems in a technique referred to as NMR Crystallography [104].

It is our belief that these differences in conformation may play a role in the toxicology of the compound, particularly regarding the interaction with the ribosome. The cellular environment is not strictly aqueous. Molecular crowding and protein active sites often result in a reduction of water availability. The peptidyl transferase center (PTC) of the ribosome is no exception [146]. During peptide bond formation, water is sequestered from the ribosome active site where trichothecenes are believed to interact. It is a possibility that the sequestration of water during peptide bond formation may serve to trigger a conformational change in the toxin, allowing for a tight binding to the PTC, thus stalling protein synthesis; while differences in the substituent groups of the trichothecene core and alternative RPL3 isoforms may contribute to the differences observed for Types I (initiation), E (elongation) and T (termination) ribosomal inhibition.

### 3.5. NMR Crystallography

NMR has been applied to crystallographic analysis for the refinement of three-dimensional structure [104] since its inception in 1948. Although NMR continues to be used as a tool in the refinement of diffraction results, NMR has further proven its ability to supply information which may be difficult or even inaccessible by diffraction methods. Solid-state NMR methods have now made sufficient advancements to the point where it is possible, in favorable cases, to solve crystal structures of molecules without the aid of single crystal diffraction data.

The basic principle behind NMR and X-ray Crystallography is the same in that the experimental data is fit to a structural model by optimizing the structural parameters. For single crystal X-ray methods the diffraction pattern is used; whereas, in NMR methods there are various options based on the chemical shielding, dipolar coupling and quadrupolar coupling parameters. In general, for natural products only the dipolar and chemical shielding parameters are relevant.

Historically speaking, chemical shielding predictions from first principles in solution NMR has had limited utility due to the large systemic errors encountered by not being able to properly account for the molecular environment in solution. In the solid-state, the immediate environment is relatively static and readily described by the unit cell and the conformations of the molecules therein. The extension of solid-state electronic structure calculations to solid-state NMR parameters has been a major breakthrough [147]. The chemical shielding parameters are predicted with unprecedented accuracy to such an extent that it is possible to use experimental values as constraints in structure optimization, as long as a suitable model can be devised. The main issue here is determining the unit cell parameters, which in any practical sense, still require powder X-ray measurements. 

There are several advantages that NMR crystallography has to offer over SCXRD. NMR Crystallography has the ability to determine hydrogen positions, and offers the ability to obtain crystal structures from microcrystalline powders. In other words there is no need to grow crystals suitable for diffraction studies. Mixtures of several polymorphs can be dealt with, and often done simultaneously, as disorder due to solvent inclusion does not pose significant complications [104]; furthermore, dynamic disorder can also be accommodated. In principle the absolute stereochemistry can be determined. 

#### 3.5.1. Hydrogen Positions

In practice, X-ray diffraction on its own cannot unambiguously define the location of hydrogen atoms. Thus, crystallographers must rely on known bonding behavior to estimate the approximate location of the protons. This can prove problematic when determining the location and angles of hydrogen bonds. Interactions of this nature are crucial to the understanding of structure-function relationships; hence the ability to determine proton positions is highly desirable.

#### 3.5.2. Powder Samples and Polycrystallinity

Solid-state NMR does not require single crystal work, although it is possible with relatively large crystals and specialized goniometer probes [148]; thus, the majority of solid-state NMR studies are performed on microcrystalline and polycrystalline powder samples, which overcomes the laborious task of producing crystals suitable for crystallographic analysis. Furthermore, atomic-level structures of gels, amorphous and glassy samples can also be obtained [104]. Polymorphic compounds can be a challenge to work within the solid-state, for both X-ray Crystallography and NMR. Although in many cases, solid-state NMR can distinguish between different crystal polymorphs, using two-dimensional correlation techniques and isotopic enrichment, for samples where isotopic enrichment is not feasible, computer-based molecular modeling must be employed. 

**Figure 12:**
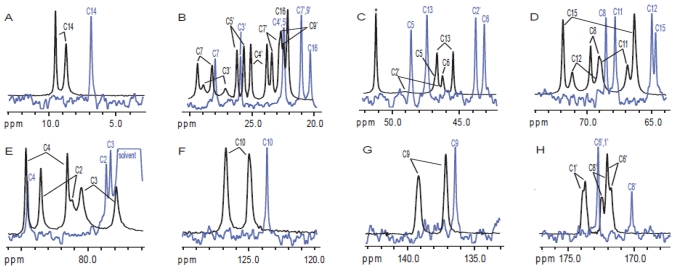
Superposition of the solid-state (black) and solution-state (blue) spectra displaying the twinning observed solid-state signals, indicating that two distinct conformations for T-2 toxin are present in the unit cell. (**A**) methyl carbons; (**B**,**C**) methylene carbons; (**D**-**G**) methine, π-bonded, and quaternary carbons; (**H**) carbonyl carbons, respectively.

Natural product, organic and pharmaceutical chemistry are laden with polymorphic compounds which may be extremely difficult to not only characterize but to quantify [149]. Solid-state NMR, and in particular NMR crystallography, is uniquely able to characterize and quantify distinct polymorphs of compounds in an efficient manner, where significant line broadening is not an interfering factor. A particularly interesting example is that of cortisone acetate [150]. The tetracyclic cortisone acetate has been previously characterized by XRD, yielding four chemical structures; however, when the compound was studied by NMR, three additional structures were determined. Each form of the compound shows a distinct peak pattern in the NMR spectra; however, in this case, it was possible to isolate the different forms from one another. In the case of T-2 toxin, studied via X-ray Crystallography by Gilardi *et al*. [116], and in the solid-state by Chaudhary *et al*. [57], the presence of two molecules with different configurations about the isovalerate function compose the unit cell (Figure 12). The two forms are present in a 1:1 ratio, giving two sets of carbon signals of equal intensity.

#### 3.5.3. Inclusion and Dynamic Disorder

Inclusion of solvent typically does not adversely affect the data, unless intermolecular dipolar coupling to the solvent results in line broadening in the spectrum. Often inclusions are dynamic, thus dipolar couplings are attenuated motion. It is possible to account for motion in the structural model and use it in the refinement of the structure [104]. In fact by making measurements over a range of temperatures it is possible to measure rates of dynamic processes and determine their thermodynamic parameters [151]. 

#### 3.5.4. Absolute Configurations

The spectra parameters are predicted on a structural model just as in SCXRD. Hence the absolute configuration about any stereogenic centre can be included in such a model and optimized to experimental data. Therefore, there is no inherent constraint on NMR crystallography in determining absolute configuration as there is in the solution phase. This has been a major weakness of solution NMR and one of the main reasons SCXRD measurements are required.

## 4. Conclusions

The structural analysis of biologically active compounds is imperative to understanding the function of the molecule. Without a well-defined three-dimensional structure, determination of potential receptors and interacting units becomes comparable to the search for a needle in a haystack. Although many three-dimensional models for various members of the trichothecene family have been proposed, physiological relevance must be considered, as multiple conformations may exist in different environments.

The search for functional interacting units remains ongoing, and the exact mechanism of interaction and toxicity of the trichothecenes remains unclear. It is the opinion of the authors, that through the combined efforts of structural analysis by solution- and solid-state NMR, NMR crystallography, and molecular dynamics simulations, as well as a biochemical investigation into the kinetic and functional activity of the compounds, that the mechanism for the toxicity of these compounds can be brought to light.
